# An Experimental Analysis to Determine the Load-Bearing Capacity of 3D Printed Metals

**DOI:** 10.3390/ma15124333

**Published:** 2022-06-19

**Authors:** Bridget Kogo, Chao Xu, Bin Wang, Mahmoud Chizari, Kazem Reza Kashyzadeh, Siamak Ghorbani

**Affiliations:** 1 Department of Mechanical Engineering, Brunel University, London UB8 3PH, UK; biddyagada@yahoo.com (B.K.); bin.wang@brunel.ac.uk (B.W.); 2 College of Mechanical Engineering, Xi’an University of Science and Technology, Xi’an 710054, China; chaoxu@xust.edu.cn; 3 School of Engineering, Physics and Computer Sciences, University of Hertfordshire, Hatfield AL10 9EU, UK; m.chizari@herts.ac.uk; 4 Department of Transport, Academy of Engineering, Peoples’ Friendship University of Russia (RUDN University), 6 Miklukho-Maklaya Street, 117198 Moscow, Russia; 5 Department of Mechanical Engineering Technologies, Academy of Engineering, Peoples’ Friendship University of Russia (RUDN University), 6 Miklukho-Maklaya Street, 117198 Moscow, Russia; gorbani-s@rudn.ru

**Keywords:** additive manufacturing, selective laser melting, mechanical properties, microstructural analysis, fracture surface analysis

## Abstract

Reverse engineering is conducted based on the analysis of an already existing product. The results of such an analysis can be used to improve the functioning of the product or develop new organizational, economic, information technology, and other solutions that increase the efficiency of the entire business system, in particular 3D printed products. Therefore, the main aim of this research is to focus on evaluation of the load-bearing capacity of already existing 3D printed metals in order to see their suitability for the intended application and to obtain their relevant mechanical properties. To this end, 3D printed metallic bars with almost square cross-sections were acquired from an external company in China without any known processing parameters, apart from the assumption that specimens No. 1–3 are printed horizontally, and specimens No. 4–7 are printed vertically. Various experiments were conducted to study microstructural characteristics and mechanical properties of 3D printed metals. It was observed that specimens No. 1–6, were almost similar in hardness, while specimen No. 7 was reduced by about 4.5% due to the uneven surface. The average value of hardness for the specimens was found to be approximately 450 HV, whereas the load-extension graphs assessed prior point towards the conclusion that the specimens’ fractured in a brittle status, is due to the lack of plastic deformation. For different specimens of the 3D printed materials, the main defects were identified, namely, lack of fusion and porosity are directly responsible for the cracks and layer delamination, prevalent in SLM printed metals. An extensive presence of cracks and layer delamination prove that the printing of these metallic bars was completed in a quick and inaccurate manner, which led to higher percentages of lack of fusion due to either low laser power, high scan speed, or the wrong scan strategy.

## 1. Introduction

Three-dimensional printing, more commonly known as Additive Manufacturing (AM), was once only an advanced form of rapid prototyping, but has now gone far beyond that in the modern industrial world. In other words, it was a revolutionary step in material science, and the achievements of scholars over the past few decades have made the AM technique as one of the most popular methods in the production of industrial components in the present era [1]. For example, a 3D-printed steel bridge in Amsterdam [2], 3D printed robot jellyfish for tracking and monitoring endangered coral reefs in the oceans [3], 3D printed components of locomotive [4], a PGA rocket engine [5], 3D printed jewelry collections [6], and other products related to different industries, including aviation, healthcare, and food [7,8,9]. AM has its roots set in the late 80s, when the company created by Charles W. Hull known as 3D Systems (one of AM’s largest companies) introduced stereolithography (SLA) to the world. Although this was a type of printing which utilized photopolymers rather than metals, it was the birthplace of 3D printing idea and allowed for the same concepts that are now used in metallic production [10]. This led to further research by Dr. Carl Deckard in 1986 when he introduced Selective Laser Sintering (SLS), and then further advancements led to the discovery of Selective Laser Melting (SLM), Direct Metal Laser Sintering (DMLS), and Electron Beam Melting (EBM). These are collectively known as powder bed fusion techniques. In 1988, Scott Crump has developed an extrusion-based system of AM known as Fused Deposition Modelling (FDM) [11]. Generally, AM has many advantages over conventional manufacturing methods. Customization is one of the most prominent advantages as clients can specify the requirements of their products and this can be easily considered in the design process. Moreover, it is also cheaper in some aspects of manufacturing such as the cheaper costs and shorter time constraints of obtaining material powder compared to solid metals [12]. In reality, low-volume production is fast, reliable, and cheaper in many cases and it is extremely attractive to many high-end luxury companies like Rolls-Royce in motorsport. In this regard, F1 teams may construct a component while the vehicle is running test laps, and car manufacturers that in limitations and on-demand can build entire parts in a lower timeframe than traditional methods, which is far more economical. Logistics and other issues in the entire manufacturing process can be tedious and time-consuming; with 3D printers, all these extra steps may be excluded due to printing on-site [13]. Metal 3D printing technology, including stainless steels [14], aluminum alloys [15], nickel-based alloys [16], titanium alloys [17,18], and cobalt-based alloys [19], is applied in medical, automobile, aerospace, and manufacturing industries due to its excellent physical properties. In the current era, 3D printing is thriving in the high-technology industries such as aerospace and satellite. For example, General Electric (GE) and defense companies such as Lockheed Martin, Boeing, and Aurora Flight Sciences are among the many companies that have adopted metal 3D printing technology to further advance their business ventures and to modernize their manufacturing approach. With current developments, the AM limitations (i.e., dimension) have are slowly receding (e.g., Aurora Flight Sciences currently can produce the entire body of an unmanned drone in one step with wingspans up to 132 ft). In addition, GE being a US conglomerate have many applications for AM such as in their jet engines and medical devices. With the complexity of manufacturing process of such components via conventional methods, e.g., a nozzle for the jet engine requiring numerous cast parts to be combined, 3D printing will allow this to be completed in one piece and has a significant reduction in the manufacturing costs [1]. Likewise, some of the 3D printing applications in the automotive industry include 3D-printed electric car and 3D-printed bus namely OLLI made by Local Motors [12], 3D-printed prototype and engine parts made by Ford [20], 3D-printed hand-tools for automotive testing and assembly made by BMW [12], and 3D-printed spare parts and prototypes made by AUDI [21].

Among the AM methods, SLM is one of the most popular metallic additive manufacturing techniques, and research in this area progresses at a very high speed (exponential function). The most important advantages of the SLM process towards other AM processes are providing greater material flexibility, better dimensional accuracy, and higher resolution [22,23]. The basic concept of this method is a high-powered laser melting powder-based metallic materials layer by layer to create components. The layers of powder are spread over each completed layer by a roller, and this process continues in repetition until the desired component is formed. The material powder that is melted for the formation of the desired component is dependent on the preferred expected material properties [24]. To achieve a successful fabrication, some important process parameters should be optimized, such as powder bed layer thickness, laser power, hatch spacing, and scanning speed affecting the mechanical behavior, microstructure, density, and surface quality of the final product. Regarding stainless steel produced by the SLM method, there are some studies focusing on powder characteristics, optimization of SLM parameters, mechanical properties, and fatigue behavior of the component. Milad et al., have investigated the effect of volumetric energy density on the mechanical properties, material characteristics, microstructural evolution, and texture of 304 L stainless steel parts manufactured via AM SLM process [25]. They claimed that the Yield Strength (YS), Ultimate Tensile Strength (UTS), and microhardness of samples produced by SLM method are higher than those of 304 L stainless steel manufactured conventionally. In addition, heat treatment resulted in the nucleation of recrystallized equiaxed grains, and it caused a decrease in the microhardness value. According to a report published by Wakshum et al. [26], the porosity, hardness, and microstructural characteristics are mostly influenced by energy density in the fabrication of 316 L stainless steel by SLM technique. In this regard, Yasa and Kruth have stated that a re-melting of SLM processed 316 L significantly reduces process-induced porosity, which makes it to be an appropriate alternative to the conventional post-treatments [27]. Yadollahi et al., have proved that the SLM process parameters, including laser power, scanning velocity, hatch spacing, and fabrication orientation, mainly affect the microstructure and mechanical strength of the component under static and cyclic loads [28]. Liverani et al., have reported the possibility of obtaining near-full density samples with elongation to failure and ultimate tensile strength higher than those obtained with conventionally processed AISI 316 L [29]. They also claimed that the binding defects, gas pores, and voids associated with residual stresses are three main solidification defects. The small defects, e.g., local embrittlement or pores, mainly occur due to cyclic loads [30,31]. Riemer et al., have assessed the fatigue behavior (crack initiation and propagation) of 316 L stainless steel produced via SLM method [32]. It was concluded that crack initiation and propagation in the High-Cycle Fatigue (HCF) regime are not significantly dependent on pores and internal stresses. Moreover, fatigue crack growth behavior is significantly influenced by solidification and microstructure. Wei et al. have conducted the elemental and microstructural examinations, tensile, and nanoindentation tests to identify the effect of material (stainless steel) ratios and laser scanning speeds on cracks and pores of parts manufactured via SLM method [33]. Based on the achievements’ Wang et al. [34], the fatigue cracks of 316 L manufactured by AM SLM method are mainly affected by microstructure and SLM process defects, such as lack of fusion and pores. Ammarullah et al., have applied computational simulations to evaluate the Tresca stress in metal-on-metal bearings with different materials, including stainless steel 316 L [35]. Gait loading has been used to reflect Tresca stress conditions more accurately in daily activities for simulating metal-on-metal hip arthroplasty. They also stated that the greater Young’s modulus of material under the same loading conditions will give a higher Tresca stress. Jamari et al., have investigated the effect of surface texturing as dimples on the wear evolution of total hip arthroplasty in the metallic materials using computational simulation [36]. They indicated that surface texturing with appropriate dimple bottom geometry on a bearing surface can extend the lifetime of hip implants. As mentioned, and found in the literature review, many scholars have attempted to estimate the material and mechanical properties of 3D printed steels. In addition, many industrial and knowledge-enterprise companies have optimized 3D printing process parameters to improve the material and mechanical properties of the metallic samples. However, there are some material problems in this method of production that have not yet been completely resolved. Experimental results have shown that these problems in the material reduce the strength and thus the efficiency of samples produced by this method in industry. Therefore, recognizing such problems in the material and understanding the relationship between them and the strength of the samples is very important. In the present research, the authors have attempted to find the relationship between the structure of the material and material defects against the load-bearing capacity. Accordingly, the authors themselves did not produce the material in the laboratory and bought the material from a Chinese trading company that is very active and leading in this field. In this case, it is assumed that these samples have been produced industrially, in the best way, and using optimized process parameters. Therefore, the reverse engineering method was used, and as a novelty, a novel algorithm was proposed based on which they tested and analyzed, and in general, they moved one step ahead of the researchers who are looking to optimize the process parameters. These samples are extracted in the best case of that company. The results of previous experimental studies for these samples have shown the widespread of layer delamination and presence of cracks. This goes to prove that the printing of these metallic bars was completed in a quick and inaccurate manner, which led to higher percentages of lack of fusion due to either low laser power, high scan speed, or the wrong scan strategy. Therefore, in the present paper, the main aim is to focus on the load-bearing capacity of 3D printed metals produced by SLM method. In this regard, microstructure characteristics, mechanical properties, and lab examinations of different types of 3D printed metals were employed under various conditions to evaluate their suitability for reproducing them with higher quality or special applications. Afterward, the obtained results were discussed in terms of defect analysis, microstructural analysis, microhardness test, mechanical properties, and fracture surface analysis. Finally, the findings of the current research were collected.

## 2. Methodology

Engineering is a profession concerned with the design, manufacture, inspection, and repair and maintenance of products, systems, and structures. The process of duplicating an existing component, subassembly, or product without the aid of drawings, documentation, or a computer model is known as Reverse Engineering (RE). RE is very common in areas as diverse as software development, entertainment, automotive, consumer products, microchips, chemicals, electronics, and industrial mechanical components. RE makes it possible to shorten the time in the manufacturing cycle as well as extract the design information from an existing product, including materials, processes, and geometric details [37]. In general, RE is considered as a powerful process analysis tool in a system for various purposes, including the following:

− Definition of the system components and their interrelationships;
− Creation representations of the system in a different form or at a higher level of abstraction;

RE is useful due to:

− Development of design documentation, working documentation, and drawings for further production;
− Obtaining an ediTable 3D model for further research and analysis on the product and its development;
− Restoration of a damaged product or part of it;
− Geometric controlling, comparison of the part with the original 3D model/drawings, and finally deviation analysis;
− Development of additional parts considering the geometry of an existing product;
− Product modernization, analysis and optimization of the resulting 3D model (analysis of strength, mass, thermal, hydraulic, and electrical characteristics);
− Development of manufacturability (simplification and cost reduction in the manufacturing process).

**Figure 1:**
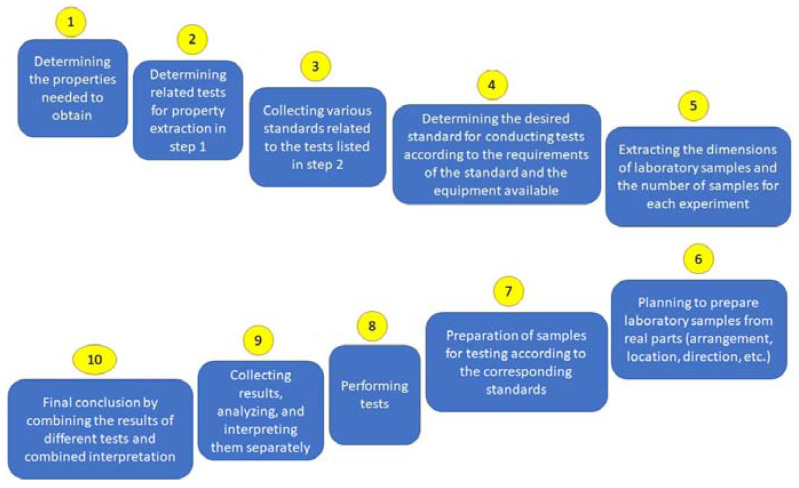
The proposed flowchart for RE of 3D printed metals with the aim of estimating the load-bearing capacity.

According to the aforementioned factors, in the present research, it is assumed that there are a series of prepared 3D printed metals, and the main aim is to identify their load-bearing capacity, mechanical properties, hardness, and microstructures. By evaluating these features, it would be possible to reproduce them or use them in different situations depending on the working conditions. To this end, the next flowchart was proposed (Figure 1).

## 3. Experimental Data

### 3.1. Specimens

**Figure 2:**
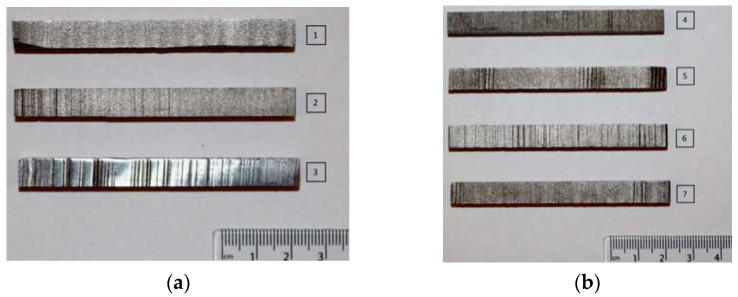
The 3D printed metallic bars: (**a**) sample No. 1–3 were printed horizontally and (**b**) sample No. 4–7 were printed vertically.

Three-dimensionally-printed metallic bars with almost square cross-section were acquired from an external company in China, without any known processing parameters apart from the assumption that three of them are printed horizontally as shown in Figure 2a, and four of them printed vertically as shown in Figure 2b. From this figure, surface of the samples is uneven, and this indicates that the samples are fabricated directly from the 3D printers without any post-processing such as surface finishing. Therefore, dimensions were initially measured (Table 1), which is 80 cm long and close to 8 mm wide and thick.

The primary inspection showed that there were signs of incomplete printing or damage during the removal of the specimens from the substrate (samples No.1 and 5, where the ends of the samples are missing material). Although the sample surfaces were very rough, a quick analysis also depicted signs of lack of fusion (e.g., in sample No. 3, there are horizontal lines of lack of fusion from the middle towards the right side of the specimen (see dotted lines)). No further defects were seen or identified at this stage. Moreover, the magnetic properties of the samples were investigated, as this property is a good indicator of the chemical composition of the metal. The results showed that all samples are magnetic. Accordingly, it was assumed that they are ferromagnetic (ferritic microstructure), thus the powder they were created from could either be an iron, nickel, or cobalt alloy. This issue will be discussed by future analysis of the experimental results (i.e., chemical composition analysis via the SEM).

### 3.2. Test Procedure

To conduct this project in the most efficient and accurate way, a step-by-step process was devised to keep on track and for time management. This section outlines these processes from post-processing to mechanical and metallurgical testing, used equipment, and the justification of these processes.

#### 3.2.1. Microscopic Observations and Defect Analysis

**Figure 3:**
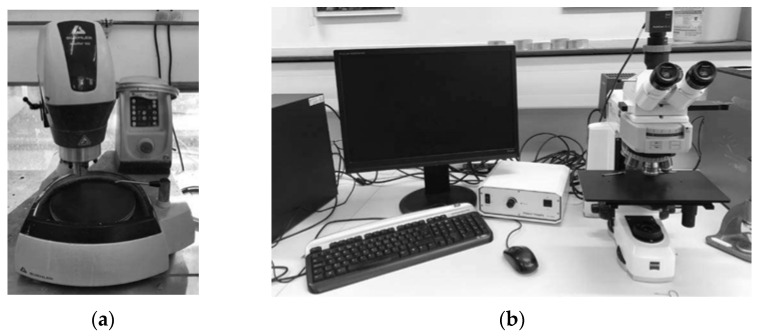
(**a**) Buehler Automet 250 Utilized for grinding and polishing processes throughout the experimental phases. (**b**) Zeiss Lens Microscope utilized in the current research with the magnification levels of 2.5× to 100×.

The SLM printed bars were acquired with no processing information; thus, extra steps were necessary before moving onto the various testing phases. For example, it is important to reduce surface roughness for performing any hardness tests, initial microscopy, and EDX spectroscopy alongside the SEM. In other words, the level of surface roughness plays a major role in the results of these tests and can affect the interpretation of the results. To this end, Buehler Automet 250 polishing machine and sandpaper with various grit sizes (P80–P4000, according to ISO standard) were used. Figure 3a demonstrates the machine utilized to obtain a polished surface for further analysis in this study. The cooling system of this device works with water. Moreover, its rotation speed is also adjustable. Further polishing was also possible using the current equipment but was not required at this step as the sandpaper alone revealed most of the defects. After polishing, the sample sizes were measured again, as these parameters are required for subsequent calculations and references. However, due to the small thickness of the samples, direct polishing was not possible, and the sample could not be guided by hand. To overcome this problem, ATM OPAL 410 resin mounting machine was used to prepare the sample. Thus, Bakelite resin was employed and the hot step for melting the resin was performed at 200 °C for 3 min. After that, the liquid resin was hardened by cooling circulating water for 3 min. Next, to appear the microstructure, according to the results of chemical composition analysis (detection of stainless steel and high percentage of chromium in the material), Kalling solution was used. This etching reagent contains 2 g Copper Chloride, 60 mL Methanol, 20 mL Water, and 40 mL Hydrochloric Acid. The action of this solution on the target metal caused to darken of ferrite and martensite phases [38]. A Zeiss Lens microscope was utilized to identify and assess the microstructural defects expanded in the previous section, including lack of fusion, porosity, cracks, and layer delamination. In addition, AxioVision software package was employed to analyze images. Figure 3b illustrates the equipment facilities used in this part of the research. This software had many features such as being able to adjust the contrast, brightness, etc., as standard and the most useful feature was the panoramic image acquisition. This allowed for entire surfaces to be documented at higher magnification levels which would make the analysis of these surfaces easier.

#### 3.2.2. SEM and EDX Spectroscopy

**Figure 4:**
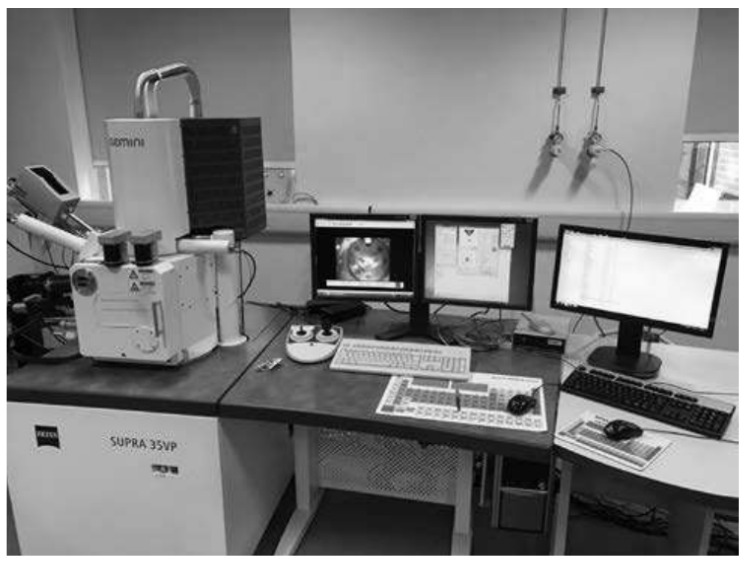
Zeiss SUPRA 35VP SEM alongside the central console utilized in the current research.

SEM observations were performed using SUPRA 35 VP (Carl Zeiss, Jena, Germany) with Inca 400 facilities for a more detailed study of the defects identified in the previous step. Figure 4 displays the set-up of the microscope. Furthermore, the attached computer was of a high processing power to process the incoming signals from the SEM. The pieces were inserted into a vacuum chamber and then the central console was utilized to change the magnification and to change the orientation of the piece to the analysis face. The corresponding EDAX TEAM software was then employed to gain the composition of each test piece. Two different methodologies were performed to ensure the accuracy of the results:

1. Secondary electron technique was utilized which only assesses the emitted X-rays from the surfaces;
2. Back Scatter Detector (BSD) was utilized which assesses the composition at a set depth for the metallic bars.

In addition to the above considerations, time scanning was set to 100 s and the analyzes were conducted on different locations to ensure the results accuracy. In this stage, the compositional analysis was conducted for samples No. 1 and 5, which are printed longitudinally and vertically, respectively.

#### 3.2.3. Microhardness Measurement

**Figure 5:**
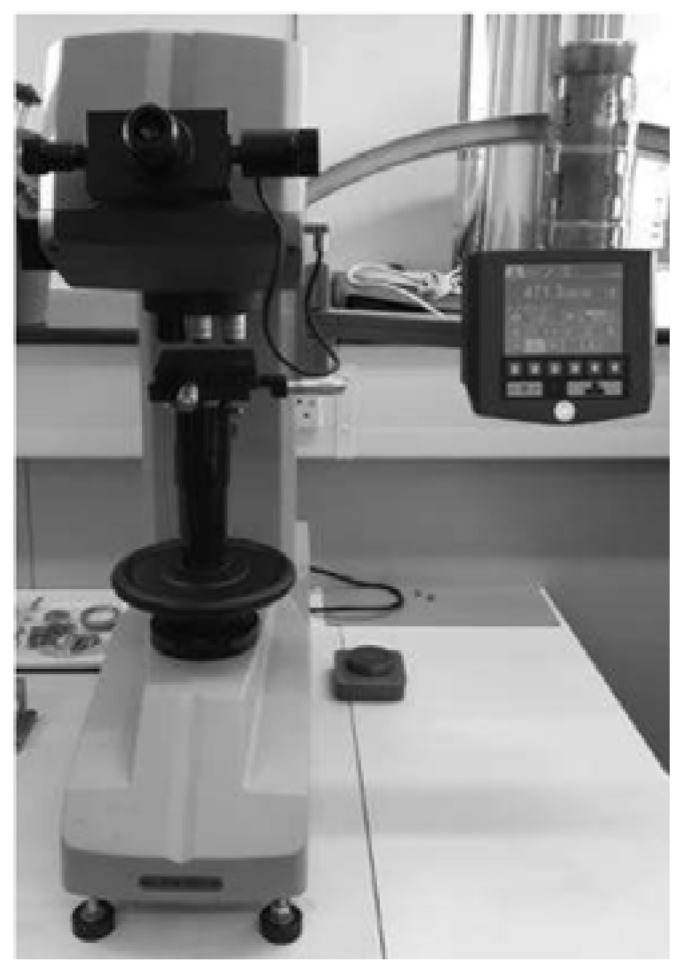
Wilson hardness testing machine utilized in the current research.

A hardness test, in addition to the material hardness assessment, is used as one of the well-known techniques for estimating the yield strength of a material without performing a tensile test (in cases where there is a shortage of material to fabricate a tensile test specimen). In most metal materials, Vickers hardness is approximately three times the yield strength in MPa [39]. Figure 5 shows the testing machine utilized to measure the hardness values at pre-determined locations. This device was calibrated before each use to ensure that the obtained values for the hardness are of a high accuracy. To this end, a diamond indenter was utilized with a load of HV10, and it was applied for a time of 10 s to create the pyramid indent, which was then scaled using the dedicated microscope to obtain a HV value for the tested location. There was a total of seven samples, and all of them were tested on two of the four faces to ensure the accuracy of the results. Moreover, 16 indents were made on each face (32 on each sample) in a straight line parallel to each other on the desired faces. Then, the mean values were used to calculate the yield strength through the above-mentioned relationship. The obtained results are also used to confirm the correlation between yield strength and hardness according to the experimental data of mechanical tests (i.e., tensile test) in future steps.

#### 3.2.4. Three-Point Flexure Testing

To extract the mechanical properties, the authors had various options for testing, such as axial tensile test, three-point flexure test, and four-point bending test. Due to the limitations of specimen dimensions (e.g., length) and compatibility with existing fixtures, the four-point bending test was omitted. Eventually, a three-point flexure test was selected. Although this test can provide a good set of results and mechanical properties, after the analysis done in this study, it was found that this type of mechanical test is not ideal due to the anisotropic behavior of metals made by 3D printing. As a result, the authors suggest for future research, axial tensile testing be performed on samples prepared in different directions of the material.

**Figure 6:**
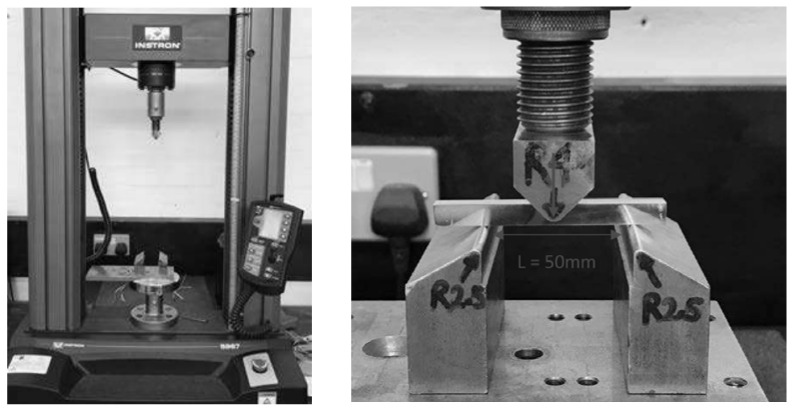
Details of the three-point flexure test performed in this research.

The fixture settings, the sample installation details, and the dimensions of the fixed and movable rollers (radius) are presented in Figure 6. In addition, the distance between the fixed rollers is equal to 50 mm and the force (P) is applied in the middle of the sample.

Three samples from each printing orientation were tested to obtain mechanical properties, including yield strength, ultimate strength, and elastic modulus. The loading was continued until the sample failed and the force–deflection diagram was recorded as the result of the three-point flexure test. Afterwards, the mechanical properties were calculated based on the test results and employing the following equations [40,41]:

(1) $$E=\frac{\sigma }{\varepsilon }$$

(2) $$\sigma_{f}=\frac{M}{I}=\frac{3M}{2tc^{2}}$$

(3) $$I=\frac{2tc^{3}}{3}$$

(4) $$\sigma_{f}=\frac{3LP}{8tc^{2}}=\frac{3LP}{2tw^{2}}$$

(5) $$\varepsilon_{f}=\frac{6wv}{L^{2}}$$

(6) $$v=\frac{PL^{3}}{48EI}$$

(7) $$E_{f}=\frac{L^{3}m}{4tw^{3}}$$

in which, E is elastic modulus, and σ and ε represent the stress and strain, respectively. Furthermore, $\sigma_{f}$ is the fracture stress, M is the bending moment, c is half of the total width, t is the thickness, I is the moment of inertia, P is the load applied on the specimen, L is the length between the rollers, v is the deflection of the beam at the midpoint, $\varepsilon_{f}$ is the flexural strain, and $E_{f}$ is the flexural modulus.

#### 3.2.5. Fracture Surface Analysis

Previous equipment was used to examine the failure surface of the samples (after mechanical testing) in both macroscopic and microscopic perspectives. To achieve this purpose, the Panorama feature was employed to analyze the images so that several magnified images could be placed side by side. In other words, by helping this feature, the entire surface was viewed and interpreted at once.

## 4. Results and Discussion

Testing is an efficient method to analyze materials under various conditions in order to study their suitability for the intended application and to obtain mechanical properties, material characteristics, etc. The mechanical properties and microstructural analysis of 3D printed metals were experimentally evaluated as described in Section 3. The obtained results were focused on the defect analysis, microstructural analysis, microhardness test, mechanical properties, and fracture surface analysis, which are presented as follows:

### 4.1. Microstructural Characteristics

#### 4.1.1. Primary Microscopic Observations and Defect Analysis

**Figure 7:**
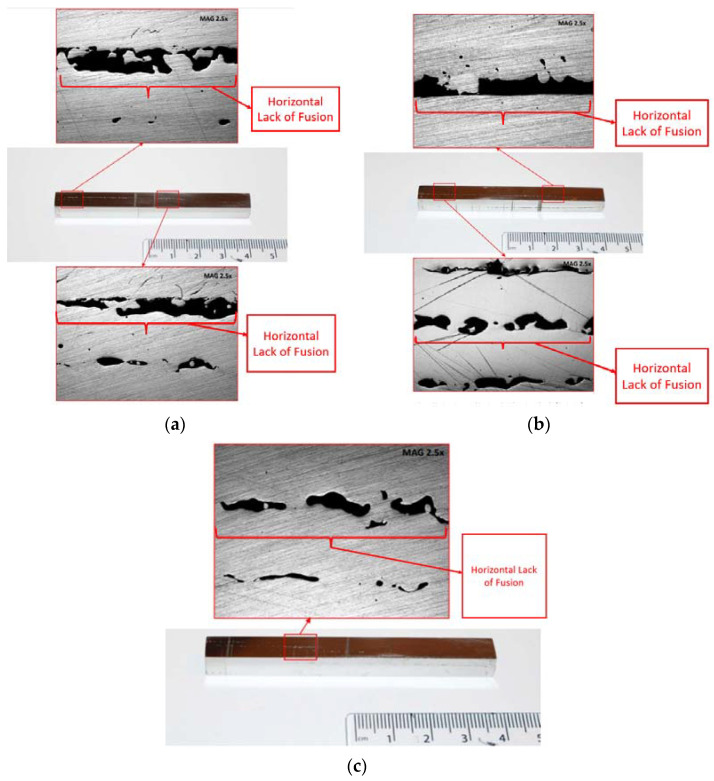
Primary microscopic observations with details of horizontal lack of fusion defects in different specimens: (**a**) specimen No. 1, (**b**) specimen No. 2, and (**c**) specimen No. 3.

**Figure 8:**
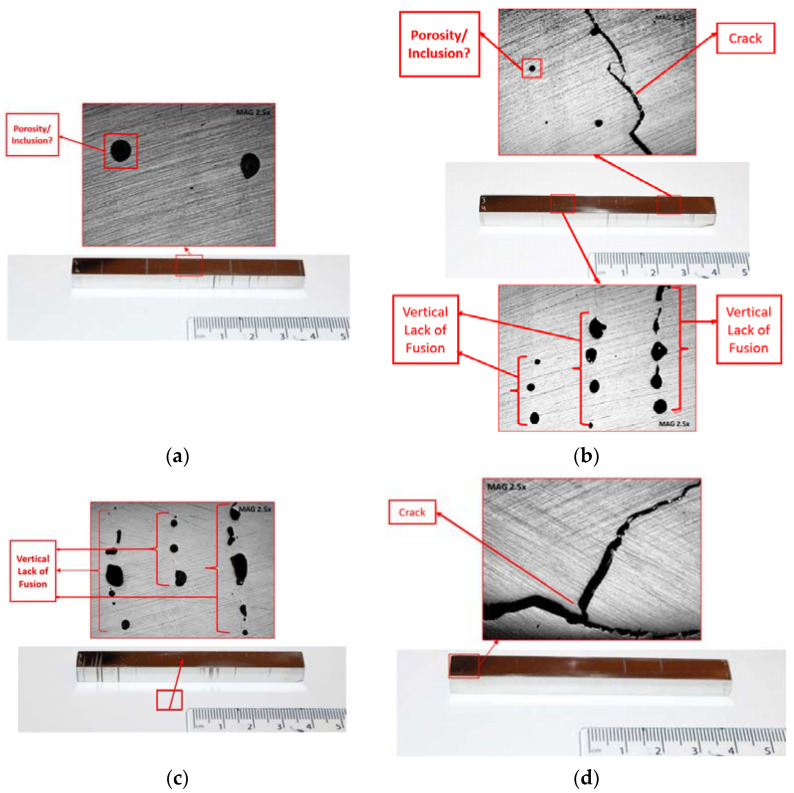
Primary microscopic observations with details of various defects such as vertical lack of fusion, porosity, inclusions, and cracks in different specimens: (**a**) specimen No. 4, (**b**) specimen No. 5, (**c**) specimen No. 6, and (**d**) specimen No. 7.

After polishing the material and reaching the mirror surfaces, most defects became visible to the naked eye such as surface cracks and lack of fusion. Afterwards, these defects were documented at a 2.5× magnification level to differentiate between the defects due to lack of fusion, porosity, and inclusions looking strikingly similar. Dimensioning was conducted again, and the results are given in Table 2. The results showed that the surface roughness decreased, and the dimensions of the specimens became more uniform compared to the raw material (e.g., the length of all specimens is currently 79 mm). Moreover, the macroscopic observations, post grinding and polishing, are displayed in Figure 7 and Figure 8 for specimens printed horizontally (No. 1–3) and vertically (No. 4–7), respectively. As observed in the initial inspection before any surface treatment, there was a great lack of fusion defect in the specimens. From these figures, specimens No. 1 and 2 displayed the highest percentage of this defect compared to other batch specimens, respectively. In addition, specimens No. 1–3 depicted the lack of fusion in the horizontal direction, whereas specimens No. 4–7 depicted this type of defect in the vertical direction. This confirmed the assumption that the samples were printed in different orientations as mentioned by the supplier. A porosity defect analysis was also conducted, but the specimens displayed little to no signs of porosity, except specimens No. 4 and 5 where, as seen in Figure 8a,b there are some signs of porosity, although these may also be lack of fusion. Both these defects (porosity and lake of fusion) can be distinguished via SEM observations. The main defect encountered in the specimens were cracks (Figure 8b,d). In specimen No. 5, the figure depicts a crack towards the center from the right side, where this crack was most probably initiated during the printing process. However, in specimen No. 7, the figure shows a much larger crack and it initiated from the edge, leading to the assumption that it was most likely generated due to improper removal from the substrate, or due to lack of care when handling the specimen. These defects may affect the flexure testing results, as lack of fusion weakens the integrity of the metallic bars, and the cracks found in specimens No. 5 and 7 may cause the results obtained for them to be inconclusive, as it can be assumed that those cracks will propagate and cause fractures alongside the intended 3-point testing fractures, invalidating the results.

#### 4.1.2. SEM and EDX Spectroscopy

**Figure 9:**
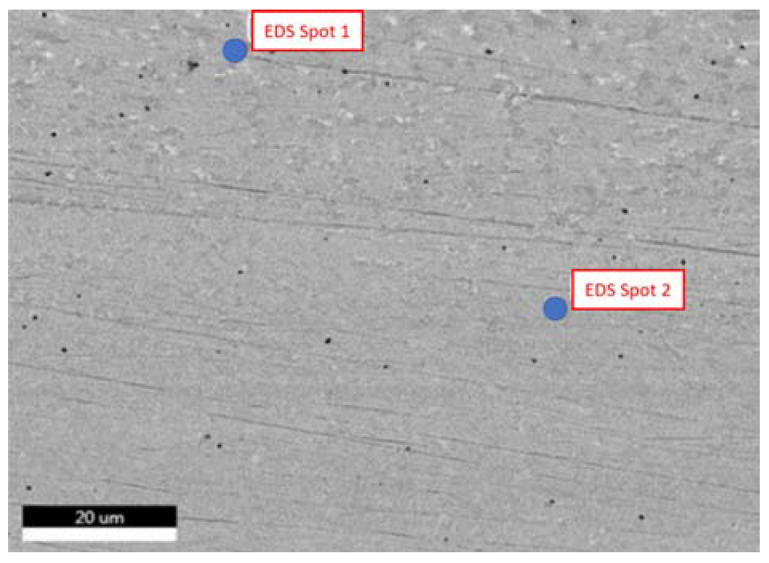
SEM image of specimen No. 1 with marked regions of compositional analysis.

After defect analysis, the samples were subjected to a SEM analysis for obtaining the composition, and to acquire high magnification images of the defects, pores, and inclusions identified in the previous step. The compositional analysis was conducted for samples No. 1 and 5, which were printed vertically and longitudinally, respectively. The analysis was conducted on different locations to ensure the results accuracy. Figure 9 shows the surface of the specimen No. 1 at the magnification of 2938×. Two points was chosen to be analyzed for the chemical composition, whereas second point was used for verification.

**Figure 10:**
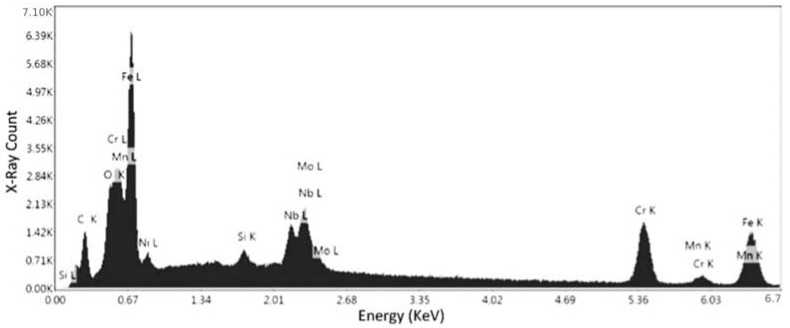
EDS measurement of the first point for specimen No. 1.

Figure 10 presents the elemental spectrum of first point for the specimen No. 1. Figure 10 and Table 3 present that there is a high percentage of iron (Fe: 33.48%) and chromium (Cr: 41.08%) in the specimen, along with a lower percentage of other elements, such as nickel and molybdenum. Due to the relatively high content of chromium, the alloy is most likely a stainless steel, which can be confirmed via the other chemical compositional analyses. Although Ni and Mn were shown to present in the composition, the error percentages for these elements were 81.51% and 44.38%, respectively. It detail, the elements and their isotopes, alongside the weight percentage at the specific point, their net intensities, the K-ratio (ratio of the intensity in the specimen to the standard element), and the Z (mean atomic number difference), A (difference in x-ray absorption), and F (difference in the production of secondary X-rays), are collectively known as the matrix corrections to differentiate the obtained specimen intensities from the standard.

**Figure 11:**
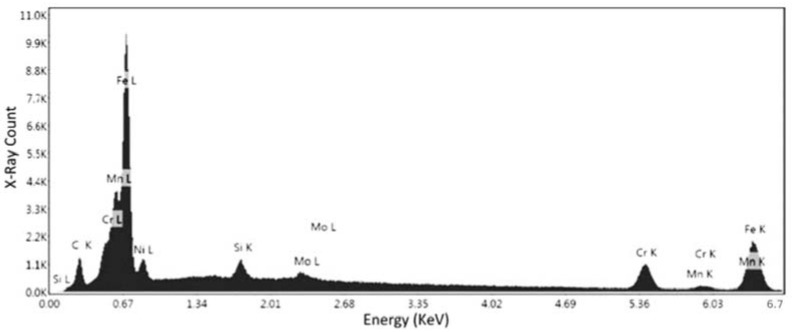
EDS measurement of the second point for specimen No. 1.

Figure 11 and Table 4 represent the confirmation elemental spectrum in the second point of specimen No. 1. The results compared to the first point show that there is still a sharp peak of iron, but there is a lower percentage of chromium in the composition. The error percentages for Ni and Mn were 19.84% and 9.40%, respectively. Although the nickel error rate is still high, the Mn error rate contradicts the results of the first point, so this needs to be confirmed. This confirms that the alloy is definitely ferrous in nature, and the weight of the chromium (29.82%) present leads the alloy towards being a stainless steel, as alloys with more than 10% chromium content are classified as stainless steels. This is the compositional analysis of specimen No. 1, which was printed in the horizontal orientation. Thus, a specimen from the second batch was also analyzed to check and confirm the inference of stainless steel for samples.

**Figure 12:**
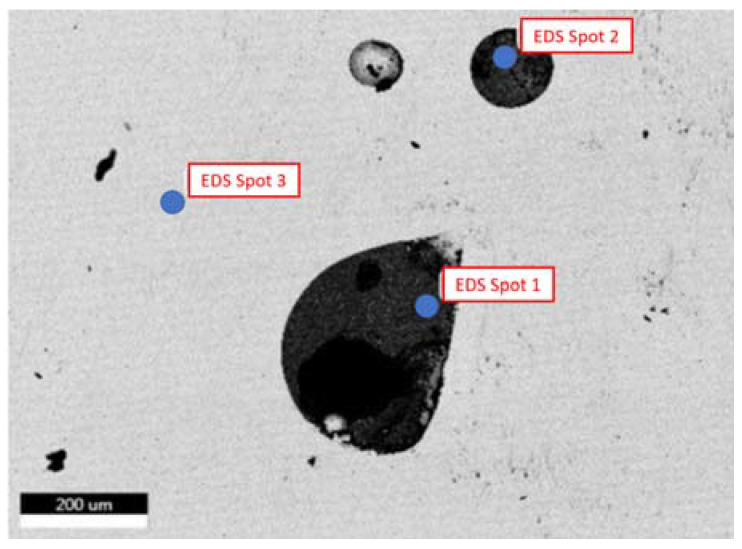
SEM image of specimen No. 5 with marked regions of compositional analysis.

**Figure 13:**
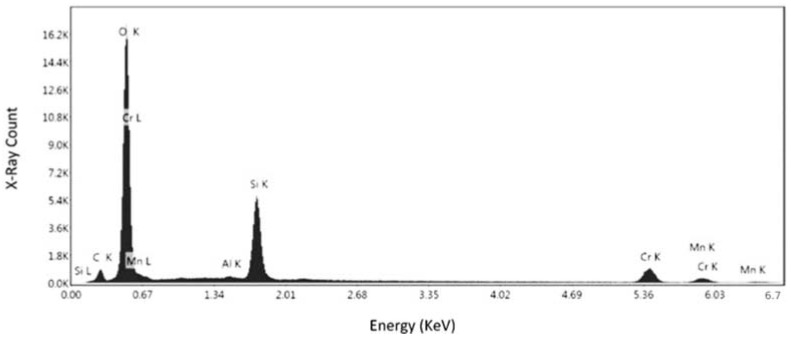
EDS measurement of the first point for specimen No. 5.

**Figure 14:**
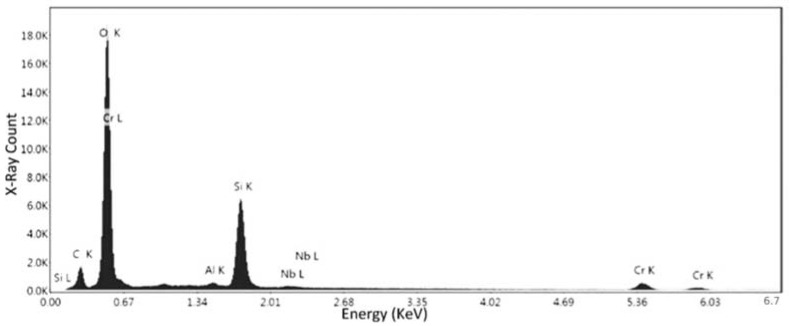
EDS measurement of the second point for specimen No. 5.

Specimen No. 5 was also analyzed using the SEM. The parameters set in this case were 10 kV for the electron beam voltage, a 250× magnification, and a scan time of 96.8 s. In addition, compositional analysis was conducted via EDX spectroscopy, and the defects were also analyzed. Figure 12 depicts the certain locations where the compositional analysis was conducted. First and second point were analyzed to assess the nature of these defects and their relevant data are available in Figure 13, Table 5 and Figure 14, Table 6, respectively.

Based on the results presented in Figure 13 (observation of oxygen peak) and the relative weight of oxygen in Table 5, which proves the presence of oxide in this location, the first point indicates the oxidation defect. Moreover, the high composition of chromium peak (Figure 13) and its relative weight percentage (Table 5) indicate that these are chromium oxides.

From the results presented in Figure 14 and Table 6, it is observed that the conditions of the second point are very close and similar to the conditions of the first point. However, there is a big difference in the relative weights of oxygen and chromium in the first and second points, but the spectrum is almost the same. Therefore, it turns out both are a kind of defect (chromium oxides).

**Figure 15:**
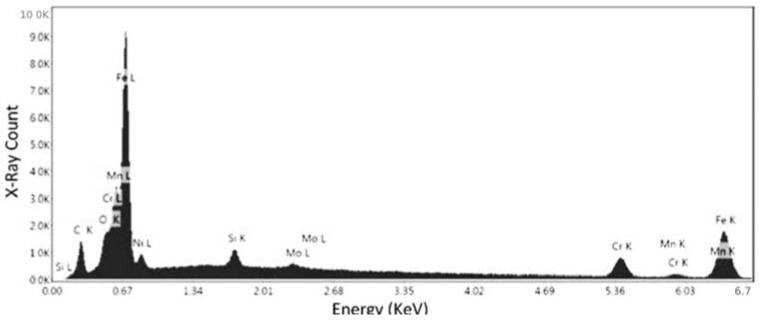
EDS measurement of the third point for specimen No. 5.

The third point analysis is to confirm the results obtained in the previous two points. As seen in Figure 15, there is visually a large amount of Fe in the chemical composition alongside Cr and Mn. Table 7 presents the data garnered from the EDX spectroscopy. From the data, the Iron (58.92%) and Chromium (25.82%) outweigh the other elements, pointing towards a chromium-iron alloy.

**Figure 16:**
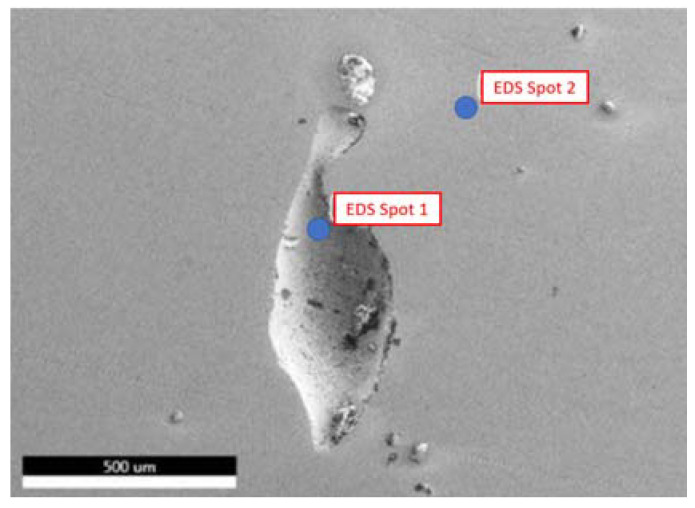
SEM image of specimen No. 5 with marked regions of compositional analysis to study the lack of fusion defect.

A second location was analyzed on specimen No. 5 using the SEM to assess the nature of this specific defect, which was hypothesized to be lack of fusion of the powders. The parameters set in this case were 10 kV for the electron beam voltage, 181× magnification, and a scan time of 96.8 s. As seen in Figure 16, two points were chosen, where the first one was directly located on the defect and the second was located on the surface. The second point was studied in order to repeat the analysis and to ensure the accuracy of the results.

**Figure 17:**
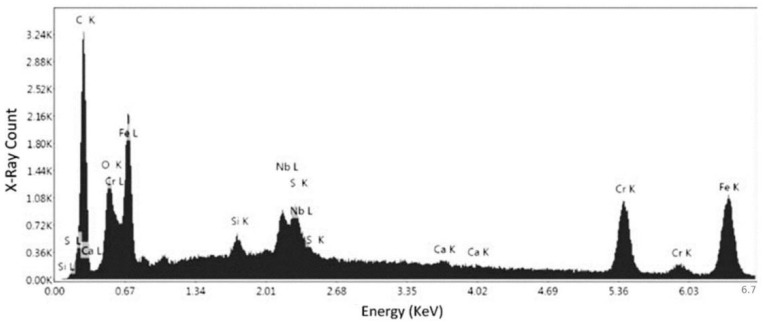
EDS measurement of the first point for specimen No. 5 to study the lack of fusion defect.

Figure 17 depicts the elemental spectrum of the first point, and the recorded data for these elements are given in Table 8. The results showed that the current defect is composed of Iron (16.6%), Carbon (24.38%), and Chromium (45.66%). This reveals that the present defect is lack of fusion, as the composition is relatively the same as the surface composition assessed on specimens No. 1 and 5. The presence of carbon in such a large quantity in this case can be disregarded, and is not due to the defect, as seen in previous analysis, the surface compositions also display a large quantity of Carbon.

**Figure 18:**
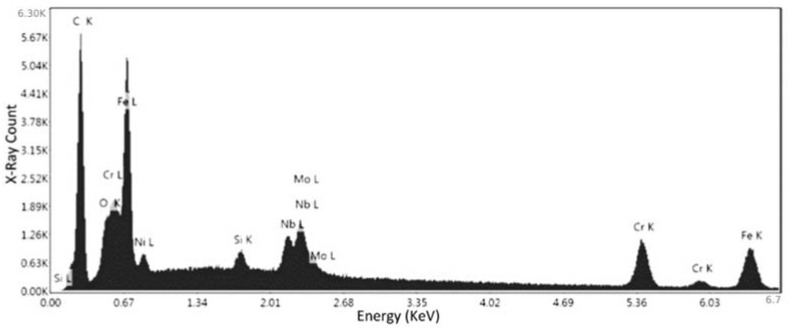
EDS measurement of the second point for specimen No. 5 to study the lack of fusion defect.

The second location on specimen No. 5 was chosen as a final check to ensure the accuracy of the compositional analysis. From Figure 18, the elemental spectrum depicts a similar trend to the previous locations, where there is a large quantity of iron and chromium. From Table 9, the relative weight of these elements is 27.07% and 30.77%, respectively. This further proves that the powder utilized in the formation was an Iron-Chromium alloy.

#### 4.1.3. Microstructural Analysis

**Figure 19:**
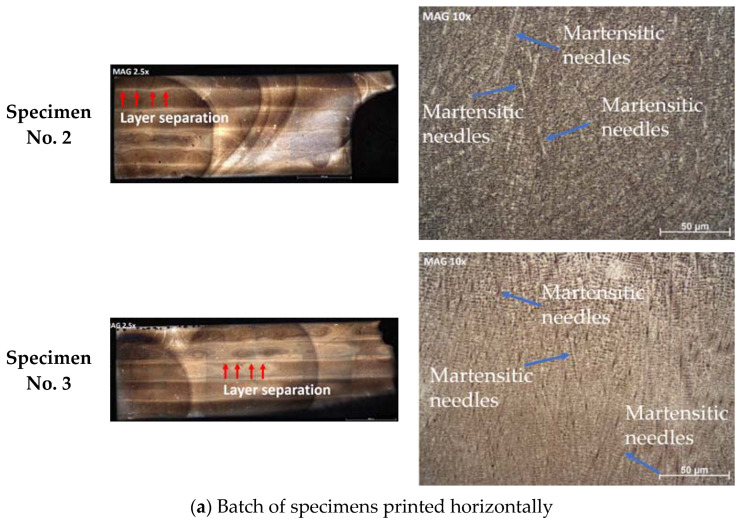

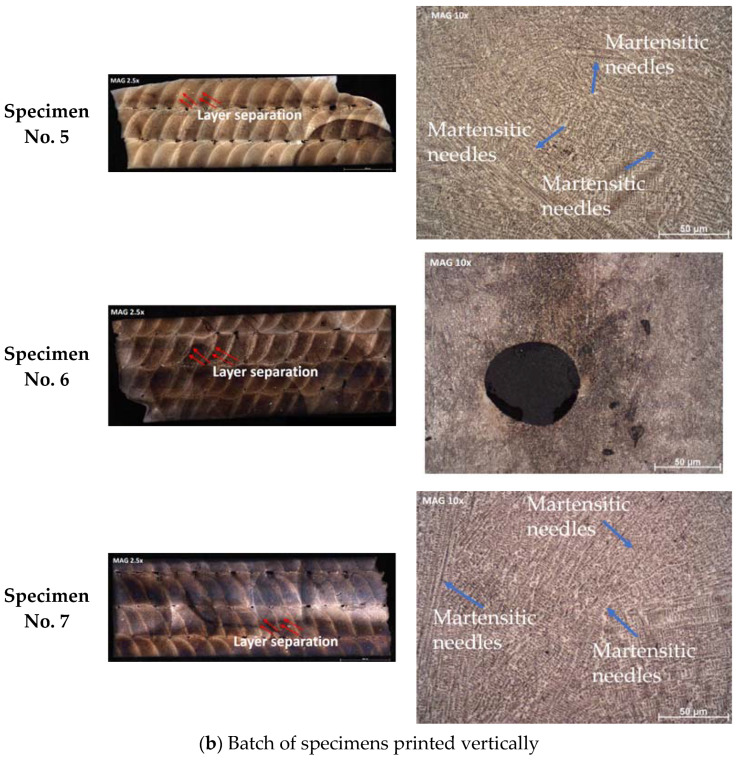
Microstructure of different batches of samples prepared by 3D printing method in various orientations, including (**a**) horizontal direction and (**b**) vertical direction.

The microstructure of tested specimens and details of their analysis are illustrated in Figure 19. From Figure 19a, it is clear that these samples were printed in the horizontal direction, and the layer separation can visibly be seen on the surface (highlighted by the red arrows). Moreover, the microstructure is unchanged around the fracture point, most likely due to the fact that brittle fracture has occurred. The layer separation is an intrinsic property of austenitic phase, while the darker locations are ferrite and martensite. Furthermore, the results show that the microstructure is composed of martensitic needles (highlighted by the blue arrows). The grain size is relatively small, which is a primary factor in brittle fractures [42,43]. When the grain size is small, dislocations are restricted before reaching the grain boundary. Due to this restricted movement, the plastic deformation is less and leads to brittle fractures. From Figure 19b, it is clear that the printing direction is vertical in this batch (highlighted by the red arrows). More defects were observed in this batch of specimens, and these defects were found at the separation of layers. This is most likely due to printing defects, such as translocation between layers, which may have reduced the laser power, or the transfer rate may have been very high. Moreover, the grain size for this batch of specimens is also very small, thus the printing direction having no effect on this feature. However, in this batch of specimens (i.e., printed vertically), there is a high concentration of martensitic needles. The last two OM images (Specimens No. 6 and 7) are darker than the other OM images. These specimens were heavily over-etched due to human errors, but the microstructural properties are still clearly visible. In analyzing these images, the ferritic nature explains the magnetic properties. Moreover, defects are seen again around the layer separation regions, which confirms that printing defects have formed with the start of the next layer. Eventually, the results of the analysis performed in this section indicate that the printing orientation does not affect the microstructural properties.

### 4.2. Microhardness Test Results

**Figure 20:**
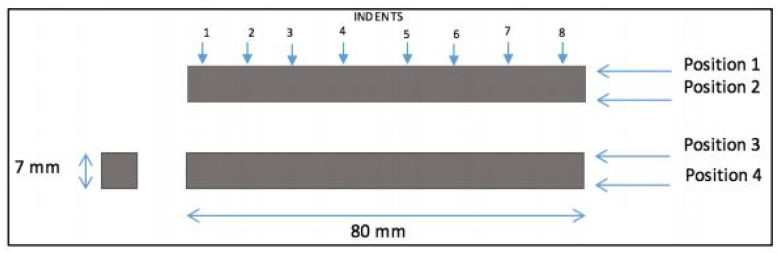
Details of different paths and the positions of indentations in the microhardness test.

**Figure 21:**
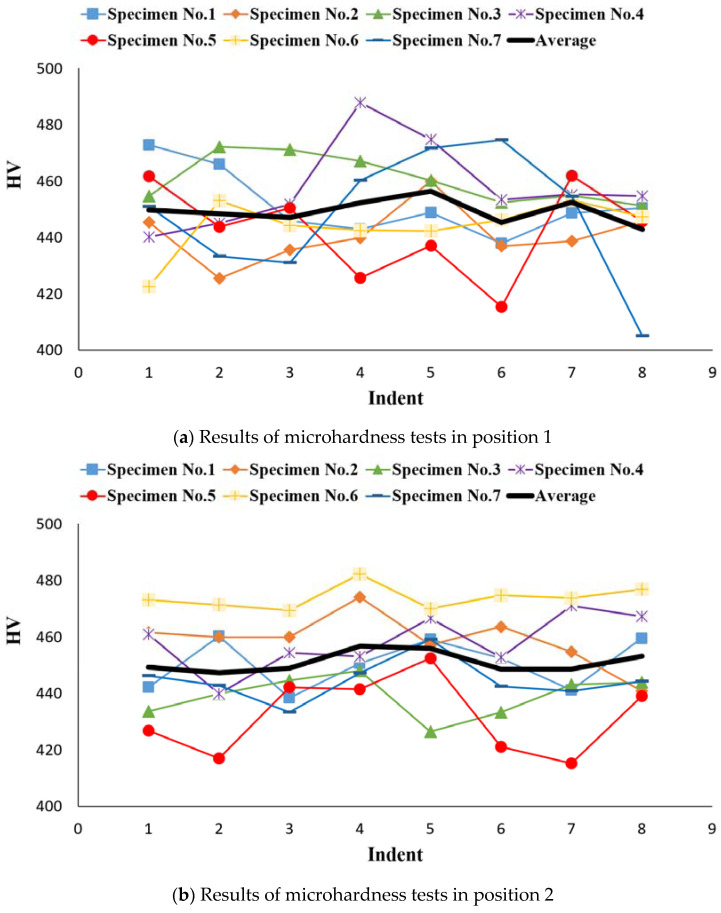

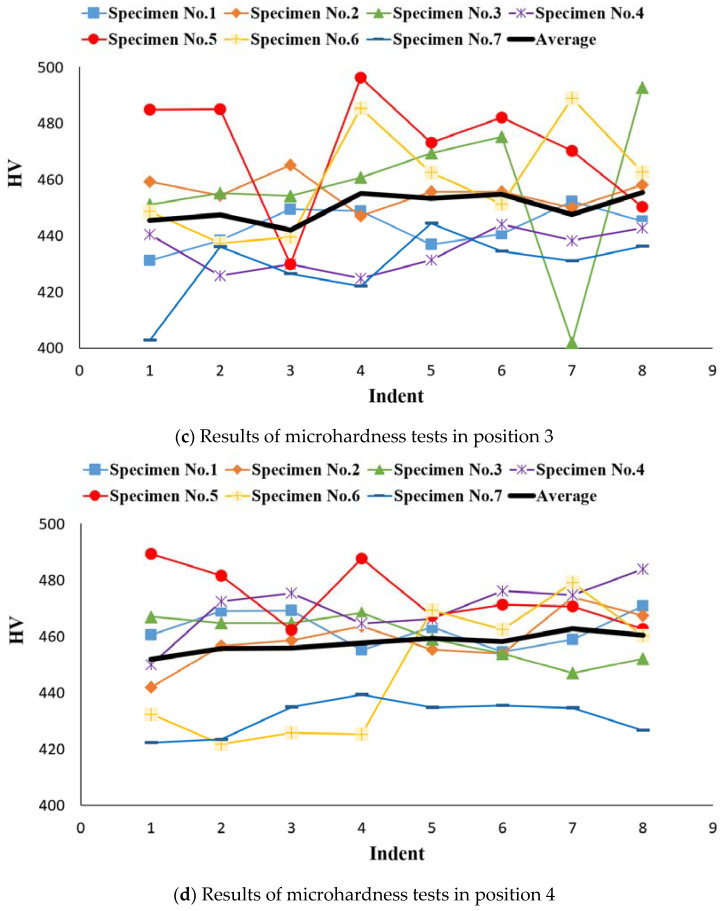
Microhardness graphs on different positions, including (**a**) position 1, (**b**) position 2, (**c**) position 3, and (**d**) position 4.

The hardness testing was conducted on two faces and four linear paths as shown in Figure 20. Moreover, eight points were tested in each path and each test was repeated twice. In other words, the mean value of the two tests indicates the hardness of that point. Figure 21 presents all the results obtained from microhardness tests for different specimens. The overall average of hardness was 452.35 HV and the yield strength was calculated to be about 150.8 MPa.

Figure 21a depicts the position values for the hardness of all specimens including both batches in position 1. Similarly, the results obtained in different positions (2, 3, and 4) are shown in Figure 21b–d, respectively. Moreover, details of the recorded values are reported in Appendix A (Table A1, Table A2). In these figures, the mean values are presented by the black line, which, in all cases, is in the value range of 450 HV. Although most of the recorded values fell within the error band, specimen No. 4 at indent 4 and specimen No. 7 at indent 8 showed a massive fluctuation compared to the other specimens. This is due to a human error in analyzing the pyramid or due to an error during test. These large variations were observed in other positions as well as in various indentation places of different specimens. Since the number of this event is small compared to the number of the performed experiments, therefore this error can be ignored.

### 4.3. Mechanical Properties

**Figure 22:**
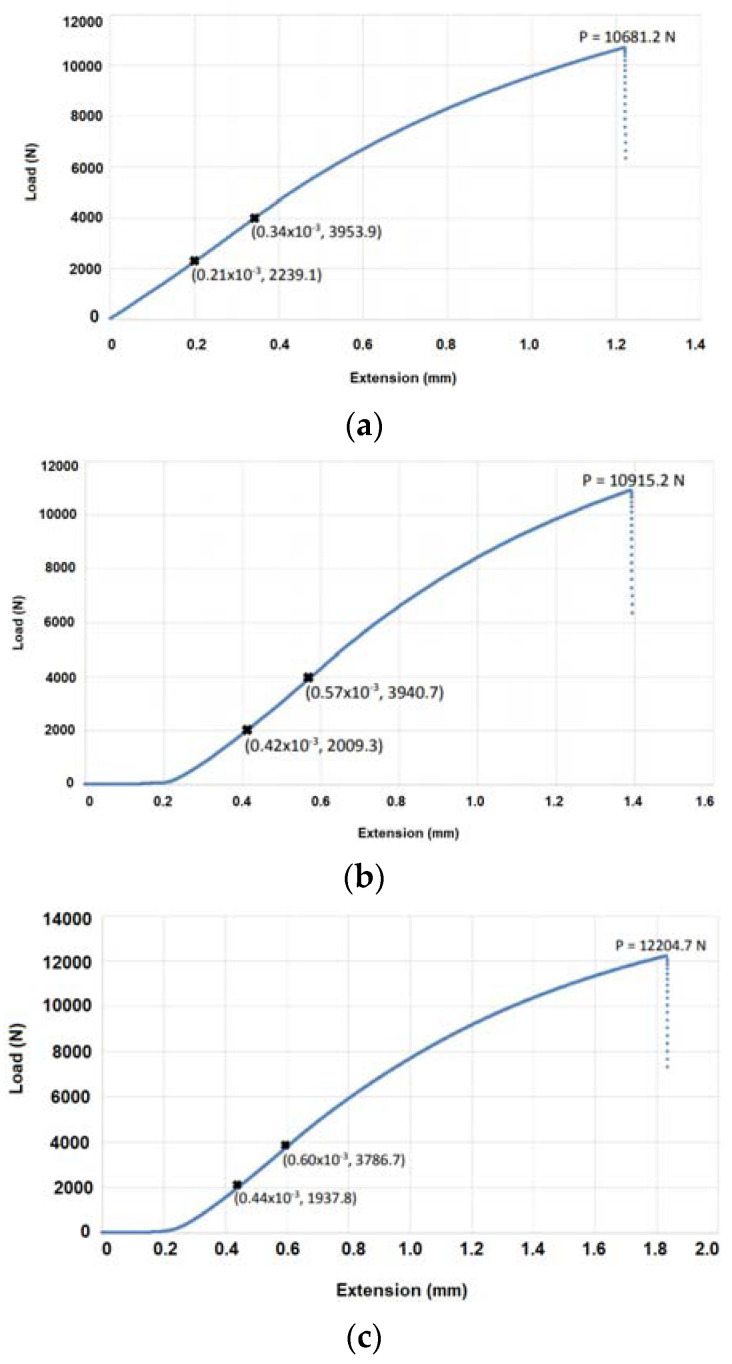

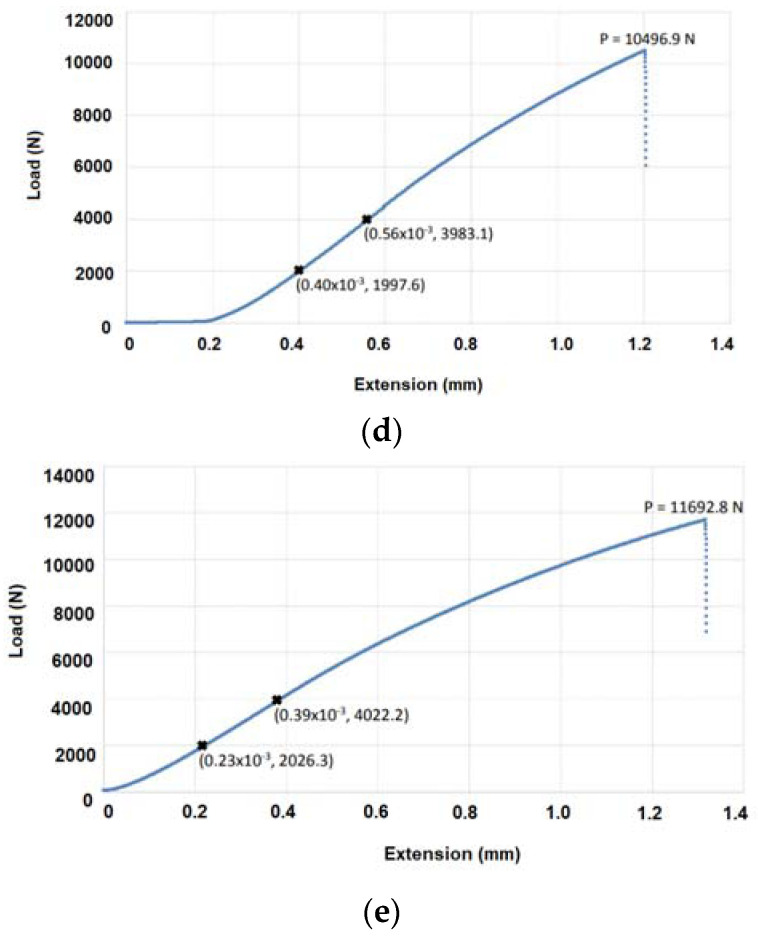
Load extension diagrams as the results of three-point flexure tests for different specimens, including (**a**) specimen No. 2, (**b**) specimen No. 3, (**c**) specimen No. 5, (**d**) specimen No. 6, and (**e**) specimen No. 7.

The results of three-point flexure tests in the form of load-deflection diagrams are demonstrated in Figure 22. From this figure, the tested specimens have a similar trend between the load and the deflection, where it starts off roughly linear and then curves towards the fracture point. In addition, there is no visible UTS on any of the graphs, the elastic deformation is minimal, the plastic deformation is also very little, and there is a very low energy absorption before the fracture point. As a result, brittle fractures occur [42]. The results of mechanical test for specimen No. 5 (Figure 22c) show that this specimen fractured at a much higher load of 12,204.7 N than the rest of the specimens. This is most likely an anomaly as this specimen fractured into 3 separate pieces, rather than 2, depicted later in the fracture surface analysis. Furthermore, specimen No. 5 had a much larger extension compared to the other results (the extension was over 1.8 mm, and the rest of specimens had an extension in the range of 1.2–1.4 mm). Moreover, another interesting observation was that specimen No. 7 was fractured under the load of 11,692.8 N, although its extension was less than specimen No. 5.

To calculate the mechanical properties, specifically the flexural stress $\sigma_{f}$ and the flexural modulus $E_{f}$, Equations (4) and (7) were utilized, respectively. The results of calculations are reported in Table 10, where it can be clearly seen that the values for specimen No. 5 are outliers. This is an error in the results obtained from the three-point flexure test for this specimen, as depicted later, the specimen fragmented into three pieces rather than two. This could be due to human errors, such as incorrect loading, or the specimen could have been weaker in the second fracture location due to the cracks identified at the defect analysis stage, thus prompting the need for future four-point bending analysis to test for a wider stress range, rather than a single point. The average flexural stress, without including the results of specimen No. 5, was calculated to be $\sigma_{f}=1667.8\;\mathrm{MPa}$ and the average flexural modulus was calculated to be $E_{f}=96.8\;\mathrm{GPa}$. These values will be compared against traditionally manufactured stainless steels in the discussion section, to conclude whether 3D printed parts can be adopted for a wider range of uses. The flexural stress calculated based on the mechanical tests was much higher than the one estimated from the hardness tests. This is due to the relationship defined for the hardness value and the yield stress being for tensile testing and not for bending tests.

### 4.4. Fracture Surface Analysis

**Figure 23:**
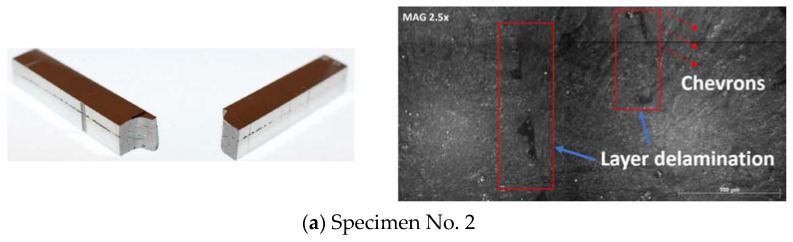

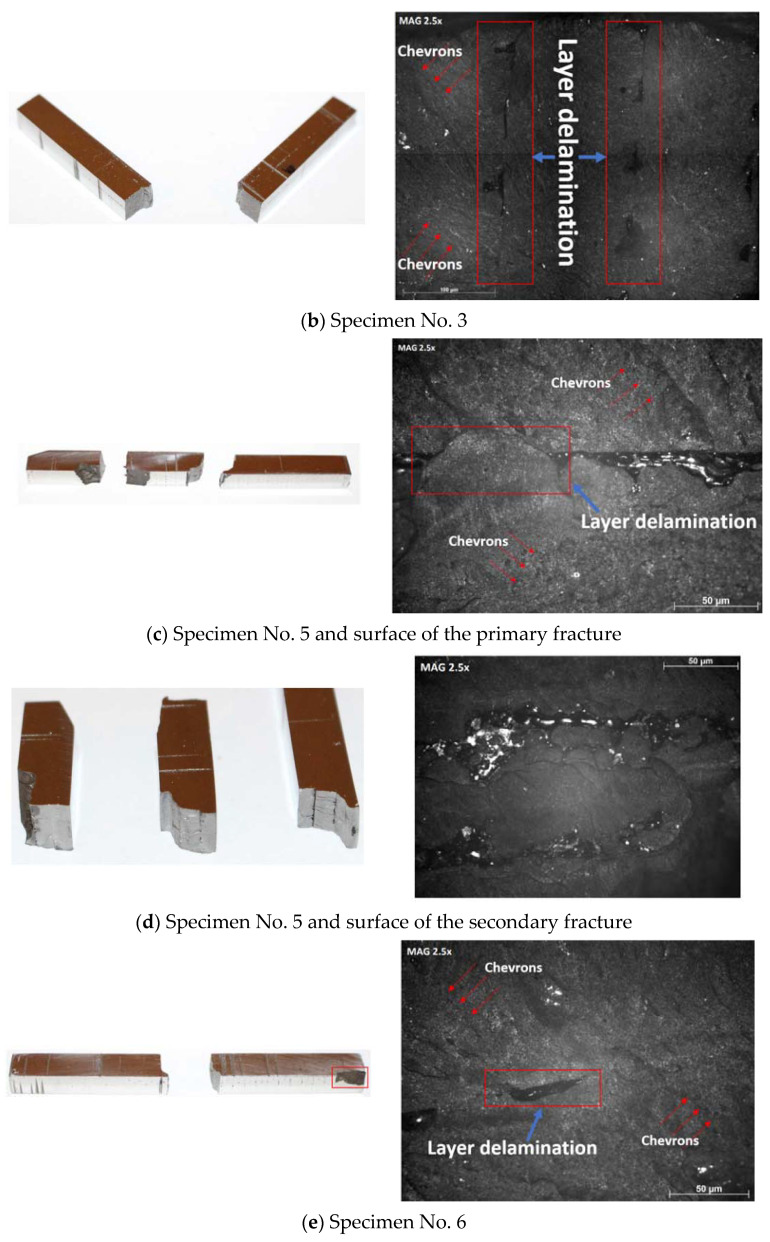
Fractured specimens and their respective surface features.

The load-extension graphs assessed prior point towards the conclusion that the specimens fractured in a brittle fashion, as there was a lack of plastic deformation. Figure 23 display the fractured specimens and their respective surface features. Figure 23a depicts the two fragmented pieces of specimen No. 2. It is clear that there is extensive layer delamination, where, visibly, there are only four layers in the specimen and the lack of fusion defects are integrated into these. The lack of fusion between the layers is the most probable cause for the layer delamination. Moreover, it is obvious that there was a clean break, with little to no plastic deformation, and visibly the surface can be seen to exhibit brittle features and is flat. In other words, all the common features in brittle fractures [42] can be seen in this specimen. To further assess the nature of the fracture and the material properties, the surface of the specimen was analyzed via SEM images. In this figure, the layer delamination can clearly be seen as marked in red boxes. The pattern on the surface, more specifically the V-shaped marks (more visible on the right side as indicated by the red arrows), are called “chevrons”. These are mainly features related to brittle fractures, thus confirming the initial assessment [42]. Figure 23b depicts the fragmented pieces of specimen No. 3. Evaluation of this sample showed the similar results to sample No. 2 (the layer delamination and the lack of fusion are interconnected). This layer delamination is most likely due to the printing method and process parameters such as high scan speeds and low laser power. Due to the parameters for these specimens not being disclosed by the manufacturer, this is only a hypothesis. SEM image of failure surface of this specimen also exhibited similar features to specimen No. 2, where there are chevrons present (red arrows), which is a common brittle fracture pattern, and the layer delamination due to lack of fusion can also clearly be seen (red boxes). The interpretation of the images related to specimen No. 5 is demonstrated in Figure 23c. It is the most interesting fracture, as the specimen fractured at two points, rather than at the one point where the stress was concentrated during the flexure test. This could be due to human errors during the apparatus set up. During the testing phase, this specimen shot off after fracturing and the impact due to high velocity could be the causality leading to the secondary fracture. The primary fracture displayed the features indicating a brittle fracture, like the previous specimen. Although an interesting observation is that the surface is not entirely flat, this points towards an intergranular brittle fracture. An intergranular fracture is when the crack propagates along the grain boundaries and continues until the fracture is complete [42]. Figure 23d depicts the surface of the primary fracture for specimen No. 5. The features exhibited here such as the chevrons patterns depicted by the red arrows, are an indicator for a brittle fracture. In addition to these issues, the surface analysis showed similarities to the previous specimens, except some interesting observations. The layer delamination wasn’t complete, as depicted by the red box in this figure; the powder seems to have melted and completed the connection of the two layers and is most likely a printing error. Figure 23e illustrates the fragmented pieces of specimen No. 6. The fracture was very similar to specimen No. 3, and it exhibited the same brittle fracture properties. An interesting feature of this specimen is the damage at the edge. This is due to the specimen shooting off from the apparatus at a high velocity after the fracture. The layer delamination was not as significant in this specimen, and also it is hard to distinguish between the layers, which was much easier in the previous specimens. This makes this specimen structurally better than the other specimens, as the lack of fusion is less and the defects surrounding these are not as significant, thus not compromising the structural integrity as much. Moreover, the fracture surface exhibited clear chevron patterns as represented by the arrows on the image.

### 4.5. The Mechanical Loading Capacity of the SLM Parts

After confirming that these metallic bars were indeed martensitic stainless steels, a comparison between the AM alloy and traditionally formed alloys was conducted. The average calculated / determined mechanical properties for these specimens were as follows:

$$Hardness=452\;\mathrm{HV};\;\sigma_{f}=1667.8\;\mathrm{MPa};\;E_{f}=96.8\;\mathrm{GPa}\;$$

Martensitic stainless steels with a similar composition, but formed via traditional methods typically exhibit the following mechanical properties [44]:

$$Hardness=480–580\;\mathrm{HV};\;\sigma_{f}=780-980\;\mathrm{MPa};\;E_{f}=215\;\mathrm{GPa}\;$$

**Figure 24:**
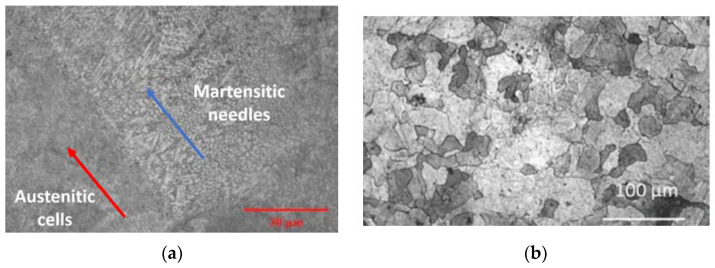
(**a**) Surface microstructure of SLM printed stainless steel directly from the substrate [43] and (**b**) SLM printed stainless steel microstructure following 1100 °C heat treatment [43].

In comparison, the hardness of the former is slightly higher and the flexure modulus doubles. However, the latter exhibits a much higher flexural strength. This is most likely due to the finer grain structure obtained from the AM techniques due to the solidification speed [45]. Moreover, the properties reported for the traditionally formed stainless steels were calculated and obtained in accordance with ISO and ASTM standards. Since the specimens were directly taken from the substrate without any post-processing treatments, this may have adversely affected the mechanical properties and explaining the difference in the above-comparison [46]. To achieve a certain mechanical property, most SLM parts undergo heat treatments to alter the microstructure, change the grain size and orientation to better suit the needs of the application [47,48]. As seen in Figure 24a, the microstructure and grain size evaluated in the previous section are similar to the results obtained for stainless steel in other studies. The visible difference being the orientations, as there are two distinct regions, marked by the red and blue arrows. This is due to the island scan strategy being applied, which melts the powders in alternative directions following each layer. In this research, the brittle nature of the metals was altered via heat treatments by increasing the grain sizes. In this regard, Figure 24b depicts the microstructure of the metal following a heat treatment at 1100 °C. It is clear that the grain size and structure have changed and are visibly much bigger in comparison. This made the structural integrity more ductile, allowing it for usage in such applications where ductility is required. The mechanical properties were also enhanced alongside the grain alteration, as they are directly related. This issue proves that the mechanical properties are highly dependent on the temperature and the final obtained microstructure. Therefore, heat treatments can be used to change the microstructure and thus significantly improve the mechanical properties [43].

In addition to temperature, the mechanical properties of AM metals are apparently highly dependent on the process parameters such as hatch angle, laser power, scan speed, layer thickness, overlap rate, and the build direction [45]. Regarding the build direction, following the mechanical properties analysis and the microstructural analysis, it can be explicitly stated that the build direction has no effect on the physical properties apart from the microstructure visibly being different. However, the overall microstructure was the same, where the layer overlap was austenitic in both cases and the rest of the microstructure was composed of ferrite and primarily martensitic needles [46]. In brief, the main defects identified (i.e., lack of fusion and porosity) are directly responsible for the cracks and layer delamination, prevalent in SLM printed metals. As depicted in the previous section, cracks were identified and the layer delamination was extensive. This issue proves that the printing of these metallic bars was completed in a quick and inaccurate manner, which lead to higher percentages of lack of fusion due to either, a low laser power, a high scan speed, or the wrong scan strategy was applied. The lack of fusion occurs primarily due to the incomplete melting of the powders during overlap, and that leads to a layer delamination, which adversely affected the mechanical properties. This could be seen, especially in specimen No. 7 where the fracture caused most of the layers to separate. Regardless of the printing direction, the delamination was prevalent in both cases; thus, this parameter has no effect on this defect either. As mentioned before, to reduce or eliminate these defects, thermal treatments must be applied, which, in turn, alters the grain structure due to recrystallization, and enhancing the mechanical properties simultaneously [46].

## 5. Conclusions

As AM progresses, so the parts will be developed. Trends predict that future technology will be far more advanced, such as being fully automated, more flexible, faster, and economical. These are only a few of the growths that will be seen in the future, thus making AM more attractive than traditional manufacturing for current leading markets thus promoting conversion. The widespread adoption of 3D printing is possible, but due to the mechanical requirements and lack of standards, this may be difficult in the near future and more research is needed. For example, extensive research is needed to optimize the laser speed and power to avoid layer delamination and porosity due to incomplete fusion. In addition, it is necessary to focus on using the correct scanning strategy, because the orientation of the grains depends entirely on this issue. Since the nature of the microstructure (martensitic), and subsequently the mechanical properties of the material, depend on the heat treatment and its details, study of time changes of heating and cooling process is highly recommended. Moreover, deposition method of the powders must be chosen wisely and tailored towards the intended use of the metals. In future research, the authors seek to investigate the behavior of 3D printing materials by SLM method under cyclic loading. Considering the studied defects and the uncertainties in different measurements, the authors believe that the relationship between the HCF behavior (S-N curve) depends on the type of loading (i.e., axial tension-compression and bending), and it is different from the relationship defined for metals made in the traditional way. In addition, in the next study, the effect of frequency and stress ratio parameters will also be studied.

## Figures and Tables

**Table 1:** Primary dimensions and magnetic properties of the samples.

Sample No.	Length (mm)	Width (mm)	Thickness (mm)	Magnetism (Y/N)
1	79.8	8.2	8.1	Y
2	79.7	8.4	8.2	Y
3	80.2	7.9	7.8	Y
4	80.0	7.7	7.9	Y
5	80.1	8.3	8.0	Y
6	80.5	8.0	8.1	Y
7	79.6	8.1	8.1	Y

**Table 2:** Dimensions of samples after post-processing.

Specimen No.	Length (mm)	Thickness (mm)	Width (mm)
1	79	7.81	8.05
2	79	7.58	7.97
3	79	7.76	7.94
4	79	7.84	7.91
5	79	7.57	7.45
6	79	7.65	7.87
7	79	7.54	8.34

**Table 3:** Chemical composition as the result of EDS analysis in specimen No. 1 (first point).

Element	Weight %	Atomic %	Net Int.	Error %	K Ratio	Z	R	A	F
C:K	6.91	24.63	91.38	9.83	0.0326	1.3760	0.8533	0.3422	1.0000
O:K	2.88	7.70	86.86	9.09	0.0200	1.3075	0.8789	0.5324	1.0000
Fe:L	33.48	25.65	263.43	7.97	0.1499	0.9763	1.0136	0.4595	0.9979
Ni:l	0.07	0.05	0.74	81.51	0.0003	0.9892	1.0276	0.4650	0.9975
Si:K	0.56	0.85	22.96	13.22	0.0053	1.1739	0.9373	0.7947	1.0071
Nb:L	5.78	2.66	97.56	6.35	0.0489	0.8600	1.0968	0.9655	1.0187
Mo:L	7.69	3.43	124.38	6.84	0.0649	0.8492	1.1003	0.9753	1.0174
Cr:K	41.08	33.81	213.28	5.70	0.4139	0.9821	0.9996	0.9944	1.0317
Mn:K	1.54	1.20	5.52	44.38	0.0153	0.9588		1.0020	0.9944

**Table 4:** Chemical composition as the result of EDS analysis in specimen No. 1 (second point).

Element	Weight %	Atomic %	Net Int.	Error %	K Ratio	Z	R	A	F
C:K	7.25	26.11	86.42	9.67	0.0356	1.3680	0.8571	0.3595	1.0000
Mn:L	3.56	2.80	26.70	9.40	0.0211	0.9543	1.0107	0.6240	0.9981
Fe:L	53.66	41.58	446.96	6.65	0.2944	0.9704	1.0179	0.5665	0.9979
Ni:l	1.97	1.45	15.49	19.84	0.0084	0.9831	1.0318	0.4329	0.9975
Si:K	1.41	2.18	48.21	9.54	0.0128	1.1663	0.9407	0.7734	1.0049
Mo:L	2.34	1.06	32.24	15.53	0.0195	0.8435	1.1040	0.9658	1.0206
Cr:K	29.82	24.82	134.30	6.38	0.3017	0.9741	1.0013	0.9979	1.0407

**Table 5:** Chemical composition as the result of EDS analysis in specimen No. 5 (first point).

Element	Weight %	Atomic %	Net Int.	Error %	K Ratio	Z	R	A	F
C:K	6.05	12.17	62.24	9.94	0.0254	1.1994	0.9232	0.3494	1.0000
O:K	37.06	55.91	1119.98	5.14	0.2943	1.1366	0.9463	0.6986	1.0000
Al:K	0.25	0.22	8.42	23.90	0.0020	0.9945	0.9886	0.7884	1.0082
Si:K	14.36	12.34	485.68	4.05	0.1265	1.0137	0.9953	0.8641	1.0059
Cr:K	31.44	14.59	122.66	5.97	0.2699	0.8304	1.0265	1.0019	1.0318
Mn:K	10.83	4.76	28.65	9.62	0.0903	0.8074	1.0236	1.0010	1.0310

**Table 6:** Chemical composition as the result of EDS analysis in specimen No. 5 (second point).

Element	Weight %	Atomic %	Net Int.	Error %	K Ratio	Z	R	A	F
C:K	13.12	20.95	117.60	9.47	0.0503	1.1244	0.9520	0.3411	1.0000
O:K	49.68	59.56	1201.08	5.86	0.3331	1.0637	0.9731	0.6269	1.0000
Al:K	0.68	0.49	21.89	10.63	0.0054	0.9281	1.0107	0.8436	1.0115
Si:K	18.14	12.38	560.58	3.61	0.1558	0.9455	1.0165	0.9042	1.0048
Cr:K	0.97	0.2	11.32	23.45	0.0068	0.6906	1.1816	0.9876	1.0175
Mn:K	17.40	6.42	60.29	6.64	0.1392	0.7695	1.0352	1.0031	1.0366

**Table 7:** Chemical composition as the result of EDS analysis in specimen No. 5 (third point).

Element	Weight %	Atomic %	Net Int.	Error %	K Ratio	Z	R	A	F
C:K	9.16	30.13	88.55	9.60	0.0448	1.3470	0.8639	0.3634	1.0000
O:K	2.03	5.01	49.03	10.34	0.0160	1.2796	0.8893	0.6162	1.0000
Fe:L	58.92	41.71	418.48	6.33	0.3383	0.9553	1.0253	0.6023	0.9979
Ni:L	0.61	0.41	3.82	64.40	0.0025	0.9677	1.0391	0.4268	0.9975
Si:K	1.42	1.99	39.08	9.49	0.0126	1.1477	0.9466	0.7736	1.0046
Mo:L	1.20	0.49	13.40	24.27	0.0099	0.8299	1.1103	0.9682	1.0211
Cr:K	25.82	19.64	94.32	6.83	0.2576	0.9566	1.0041	0.9987	1.0440
Mn:K	0.84	0.61	2.12	64.97	0.0083	0.9332	1.0057	0.9988	1.0560

**Table 8:** Chemical composition as the result of EDS analysis in specimen No. 5 (first point) to study the lack of fusion defect.

Element	Weight %	Atomic %	Net Int.	Error %	K Ratio	Z	R	A	F
C:K	24.38	55.76	204.65	8.71	0.1208	1.2623	0.8940	0.3924	1.0000
O:K	4.06	6.98	61.99	9.48	0.0236	1.1981	0.9185	0.4843	1.0000
Fe:L	16.60	8.17	68.47	9.13	0.0645	0.8941	1.0579	0.4357	0.9979
Si:K	0.20	0.19	4.77	60.52	0.0018	1.0722	0.9721	0.8359	1.0080
Nb:L	5.01	1.48	48.56	7.09	0.0400	0.7847	1.1345	0.9984	1.0205
S:K	2.93	2.51	53.70	7.8	0.0289	1.0461	0.9857	0.9315	1.0132
Ca:K	1.16	0.79	10.02	25.47	0.0122	1.0006	1.0072	0.9837	1.0697
Cr:K	45.66	24.12	130.34	5.95	0.4151	0.8861	1.0161	0.9996	1.0265

**Table 9:** Chemical composition as the result of EDS analysis in specimen No. 5 (second point) to study the lack of fusion defect.

Element	Weight %	Atomic %	Net Int.	Error %	K Ratio	Z	R	A	F
C:K	27.63	62.47	375.34	8.36	0.1403	1.2675	0.8888	0.4005	1.0000
O:K	2.51	4.26	55.17	10.44	0.0133	1.2034	0.9135	0.4404	1.0000
Fe:L	27.07	13.17	207.13	7.54	0.1237	0.8981	1.0523	0.5096	0.9979
Ni:L	0.23	0.11	2.32	66.03	0.0011	0.9097	1.0659	0.5347	0.9975
Si:K	0.80	0.77	30.15	9.76	0.0072	1.0778	0.9678	0.8322	1.0072
Nb:L	4.84	1.42	74.11	6.66	0.0387	0.7889	1.1299	0.9934	1.0189
Mo:L	6.14	1.74	89.71	6.21	0.0487	0.7788	1.1329	1.0008	1.0175
Cr:K	30.77	16.07	140.50	5.98	0.2834	0.8925	1.0142	0.9980	1.0340

**Table 10:** Calculated mechanical properties for different specimens.

	Specimen No. 2	Specimen No. 3	Specimen No. 5	Specimen No. 6	Specimen No. 7
Flexural stress (MPa)	1664	1673	2190	1662	1672
Flexural modulus (GPa)	98	98	113	105	86

## Data Availability

The data that support the findings of this study are available from the corresponding author upon reasonable request.

## Appendix A

**Table A1:** Hardness tests of specimens No. 1-3 horizontally printed.

Initial Hardness Tests												
	Specimen No. 1				Specimen No. 2				Specimen No. 3			
	Position				Position				Position			
Indent	1	2	3	4	1	2	3	4	1	2	3	4
1	472.8	443.2	430.3	460.2	446.9	461.8	459.7	442.4	455.9	434.5	452.1	467.6
2	466.2	460.3	438.8	469.9	426.2	460.3	455	455.9	471.8	439.7	455.9	465
3	447.1	439.3	448.7	469.4	437.1	460.9	464.4	458.1	471.9	444.8	454.1	465.6
4	444	450.8	448	455.2	440.6	474.8	447.9	464.6	468.4	448.3	461.4	469
5	449.4	459.2	436.7	463.4	460.2	457.4	455.3	455.8	460.3	427	469.2	460.9
6	438.5	453.8	439.9	454.7	436.8	463.3	455.5	454.4	452.6	433.8	475.5	455.3
7	449.5	441.3	451.9	459.7	439	455.5	449.1	475.3	455.8	443.9	402.4	447.4
8	451.3	460.2	444.5	471.3	445.2	441.3	457.2	468.5	450.8	443.8	492.8	452.8
Position Average	452.4	451.1	442.4	462.9	441.5	459.4	455.5	459.4	460.9	439.5	457.9	460.5
Sample Total Average	452.17				453.95				454.69			

**Table A2:** Hardness tests of specimens No. 4-7 vertically printed.

Initial Hardness Tests																
	Specimen No. 4				Specimen No. 5				Specimen No. 6				Specimen No. 7			
	Position				Position				Position				Position			
Indent	1	2	3	4	1	2	3	4	1	2	3	4	1	2	3	4
1	441.2	460.1	439.8	450.1	463.4	427.1	484.7	489.6	424	474	448.6	432.7	451.4	445.9	403.6	422.4
2	446.2	439.5	426.7	472.8	445.6	416.9	484.3	481.2	454.3	471.6	437.3	421.8	434.3	442.9	435.9	423.9
3	452	454	429.4	475.6	450.8	441.2	428.9	461.7	445.2	469.8	439.1	425.6	432.9	432.8	426.3	435.1
4	487.2	453.1	424.7	465.3	426.2	440.4	495.9	488.1	442.8	482.5	485.7	425.2	461.8	446.9	422.2	440.1
5	475.7	466.9	432.2	467	437.6	453.2	473.7	467.2	442.7	469.2	462.4	469.3	472.8	460.3	444	435.1
6	454.3	452.7	444.8	476.3	417.7	420.3	481.7	471.6	446.2	475	451.4	462.4	474.9	442.2	435.1	435.2
7	455.2	471.1	437.8	474.6	463.2	415.5	469.5	471.6	451.3	474.1	489	479.5	455.3	440.9	431	435.1
8	455.5	466.9	442	484.4	446	439.1	450.8	463.6	448.4	477.1	462.4	460.3	407.2	445.3	435.9	427
Position Average	458.4	458.1	434.7	470.8	443.8	431.7	471.2	474.3	444.4	474.2	459.5	447.1	448.8	444.7	429.3	431.7
Sample Total Average	455.5				455.3				456.3				438.6			
